# Defective AMPK regulation of cholesterol metabolism accelerates atherosclerosis by promoting HSPC mobilization and myelopoiesis

**DOI:** 10.1016/j.molmet.2022.101514

**Published:** 2022-05-10

**Authors:** Man K.S. Lee, Olivia D. Cooney, Xuzhu Lin, Shaktypreya Nadarajah, Dragana Dragoljevic, Kevin Huynh, Danise-Ann Onda, Sandra Galic, Peter J. Meikle, Thomas Edlund, Morgan D. Fullerton, Bruce E. Kemp, Andrew J. Murphy, Kim Loh

**Affiliations:** 1 Division of Immunometabolism, Baker Heart and Diabetes Institute, Melbourne, Australia; 2 Department of Diabetes, Monash University, Melbourne, Australia; 3 Department of Cardiometabolic Health, University of Melbourne, Melbourne, Australia; 4 Diabetes and Metabolic Disease, St. Vincent's Institute of Medical Research, Fitzroy, Australia; 5 Metabolomics Laboratory, Baker Heart and Diabetes Institute; Melbourne, Australia; 6 Protein Chemistry and Metabolism, St. Vincent's Institute of Medical Research, Fitzroy, Australia; 7 Umeå Centre for Molecular Medicine, Umeå University, Umeå, Sweden; 8 Betagenon AB; Västra Strandgatan 9B, 903 26, Umeå, Sweden; 9 Department of Biochemistry, Microbiology and Immunology, Faculty of Medicine, Centre for Infection, Immunity and Inflammation, Centre for Catalysis Research and Innovation, University of Ottawa, Ottawa, Canada; 10 Mary MacKillop Institute for Health Research, Australian Catholic University, Melbourne, Australia; 11 Department of Medicine, University of Melbourne, Melboourne, Australia

**Keywords:** AMPK, Atherosclerosis, HMG-CoA reductase, Cholesterol, HSPCs

## Abstract

**Objectives:**

Dysregulation of cholesterol metabolism in the liver and hematopoietic stem and progenitor cells (HSPCs) promotes atherosclerosis development. Previously, it has been shown that HMG-CoA-Reductase (HMGCR), the rate-limiting enzyme in the mevalonate pathway, can be phosphorylated and inactivated by the metabolic stress sensor AMP-activated protein kinase (AMPK). However, the physiological significance of AMPK regulation of HMGCR to atherogenesis has yet to be elucidated. The aim of this study was to determine the role of AMPK/HMGCR axis in the development of atherosclerosis.

**Methods:**

We have generated a novel atherosclerotic-prone mouse model with defects in the AMPK regulation of HMGCR (*Apoe*^*−/−*^*/Hmgcr KI mice).* Atherosclerotic lesion size, plaque composition, immune cell and lipid profiles were assessed in *Apoe*^*−/−*^ and *Apoe*^*−/−*^*/Hmgcr KI* mice.

**Results:**

In this study, we showed that both male and female atherosclerotic-prone mice with a disruption of HMGCR regulation by AMPK (*Apoe*^*−/−*^*/Hmgcr KI* mice) display increased aortic lesion size concomitant with an increase in plaque-associated macrophages and lipid accumulation. Consistent with this, *Apoe*^*−/−*^*/Hmgcr KI* mice exhibited an increase in total circulating cholesterol and atherogenic monocytes, Ly6-C^hi^ subset. Mechanistically, increased circulating atherogenic monocytes in *Apoe*^*−/−*^*/Hmgcr KI* mice was associated with enhanced egress of bone marrow HSPCs and extramedullary myelopoiesis, driven by a combination of elevated circulating 27-hydroxycholesterol and intracellular cholesterol in HSPCs.

**Conclusions:**

Our results uncovered a novel signalling pathway involving AMPK-HMGCR axis in the regulation of cholesterol homeostasis in HSPCs, and that inhibition of this regulatory mechanism accelerates the development and progression of atherosclerosis. These findings provide a molecular basis to support the use of AMPK activators that currently undergoing Phase II clinical trial such as O–3O4 and PXL 770 for reducing atherosclerotic cardiovascular disease risks.

## Highlights

• AMPK regulation of HMGCR is critical for the control of endogenous cholesterol synthesis in HSPCs.
• AMPK-HMGCR signaling regulates HSPCs mobilization and myelopoiesis.
• Perturbation of AMPK regulation of HMGCR accelerates the development and progression of atherosclerosis.

## 1 Introduction

Atherosclerotic cardiovascular disease, which stems from dyslipidemia and maladaptive inflammatory responses, precedes and predicts the development of cardiovascular complications including stroke and myocardial infarction [[1], [2], [3], [4]]. One of the key contributors to the development and progression of atherosclerosis both in animal models and in humans is the increase in circulating cholesterol levels, which is predominantly produced in the liver [[5], [6], [7]]. Elevated cholesterol can influence the function of hematopoietic stem and progenitor cells (HSPCs) and their propensity to differentiate into monocytes and other immune cells through a process known as myelopoiesis [[8], [9], [10], [11]]. Although it has been suggested that impaired cholesterol regulation in HSPCs play an important role in atherogenesis [[8], [9], [10], [11]], the molecular mechanisms that regulate cholesterol homeostasis in HSPCs during the onset and progression of atherosclerosis remain unclear. In the setting of atherosclerotic cardiovascular disease, defective cholesterol efflux in HSPCs results in enhanced proliferation and mobilization of HSPCs that reside in the BM and consequently increased myelopoiesis [[8], [9], [10], [11]]. This in turn increases atherogenic monocyte counts in the circulation, monocyte accumulation and subsequent differentiation of atherogenic monocytes into inflammatory macrophages, accelerating the development of atherosclerotic inflammation and plaque formation [[8], [9], [10], [11], [12]].

AMPK is a highly conserved key regulator of whole-body energy metabolism, including lipid and glucose metabolism, protein synthesis and mitochondrial biogenesis [[13], [14], [15], [16]]. AMPK is present in all tissues as a heterotrimeric complex composed of a catalytic α subunit (α1, α2), a scaffolding β subunit (β1, β2) and a nucleotide-binding γ subunit (γ1, γ2, γ3) and is typically inactive unless phosphorylated on Thr172 in the α subunit activation loop by upstream kinases including liver kinase B1 (LKB1) and calcium/calmodulin dependent protein kinase kinase 2 (CaMKK2) (reviewed in [[13], [14], [15], [16]]). AMPK is activated by metabolic stresses or hormonal changes that signal low energy conditions and acts to inhibit ATP-consuming anabolic processes and promote ATP-generating catabolic pathways [[13], [14], [15], [16]]. The responses induced by AMPK activation are typically short-term, involving phosphorylation of key metabolic enzymes, and long-term, which occurs through modulation of gene transcription [[13], [14], [15], [16]]. Previous studies have demonstrated that the suppression of AMPK activity under conditions of chronic over-nutrition may contribute to the development of metabolic diseases [[13], [14], [15], [16]]. In the context of atherosclerosis, AMPK has been shown to have multiple anti-atherogenic effects through its influence on inflammatory signalling [17], suppressing monocyte to macrophage differentiation [18,19], and increasing cholesterol efflux [20,21], which in combination, decreases foam cell formation and atherogenesis. In line with this, systemic infusion of direct or indirect AMPK activators such as A769662, salsalate or metformin has been shown to decrease lesion size in atherosclerotic prone mice [[22], [23], [24], [25]].

The 3-hydroxy-3-methylglutaryl coenzyme A (CoA) reductase (HMGCR) is the rate-limiting enzyme of the mevalonate pathway that is responsible for the synthesis of important nonsterol isoprenoids and bioactive sterols, such as cholesterol and steroid hormones [5,6]. HMGCR is subject to regulation at multiple levels, involving feedback control and cross-regulation by distinct biochemical pathways [5,6]. HMGCR enzyme activity is inhibited by phosphorylation at Ser871 (human HMGCR Ser872) by AMPK [26,27]. Previous *in vitro* studies have shown that metabolic stress inhibits cholesterol synthesis but not in cells transfected with HMGCR in which Ser871 has been mutated to Ala [28]. We have recently used a mouse model in which the AMPK phosphorylation site Ser871 on HMGCR was mutated to Ala (*Hmgcr KI* mice) and demonstrated that this phosphorylation event inhibits cholesterol synthesis *in vivo* and is important for suppressing the development of hepatic steatosis in response to high carbohydrate feeding [29]. While HMGCR regulation by AMPK is important for controlling hepatic cholesterol synthesis, the physiological significance of the AMPK-HMGCR axis in the development of atherosclerosis has yet to be elucidated.

## 2 Materials and methods

### 2.1 Animals

All animal care and experiments were approved by St. Vincent's Hospital (Melbourne, Australia) and the Alfred Medical Research Education Precinct Animal Ethics Committee, and conducted in accordance with the National Health and Medical Research Council of Australia's (NHMRC) guidelines for the Ethical and Humane Use of Animals in research. *Hmgcr KI* mice were generated and genotyped as described previously [29]. *Apoe*^*−/−*^ mice were obtained from the Animal Resource Centre (Canning Vale, WA, Australia). *Apoe*^*−/−*^*/Hmgcr KI* mice were generated by crossing male *Apoe*^*−/−*^ with female *Hmgcr KI* mice in a two-step manner. Mice were genotyped by Transnetyx Inc (Cordova, Tennessee, USA). All mice used for experiments were on a C57BL/6J background. Mice were housed under a controlled temperature of 22 °C and a 12-hour light cycle (lights on from 7 am to 7 pm) with *ad libitum* access to water and a standard chow diet (6% fat, 29% starch, #102108, Barastoc, Ridley Agriproducts) for 12 weeks starting from 8 weeks of age.

### 2.2 Assessment of atherosclerotic lesions

Aortic atherosclerotic lesions in the aortic root were analysed on 6um frozen sections.

#### 2.2.1 Lesion size

Sections were fixed (4min, 10% neutral buffered formalin), washed in PBS (4min), stained in Mayer's Haematoxylin (15min) and washed with running tap water before blueing in Scott's tap water for 30secs. The slides were then put in 95% ethanol (10 dips), stained in buffered alcoholic eosin (8min), dehydrated in absolute ethanol, cleared with xylene and coverslips were mounted using depex. Sections were imaged on the Olympus FSX100 microscope 4.2x magnification and images were analysed using Adobe Photoshop CC.

#### 2.2.2 Lipid content

Sectioned lesions were fixed in 10% buffered formalin (4mins), washed in PBS (4min), dipped in 60% isopropanol before staining in 60% ORO working solution (2hrs, stock solution: 1% ORO powder in isopropanol). The slides were then washed in 60% isopropanol and distilled water. Sections were stained in Mayer's Haematoxylin (4mins), washed in tap and distilled water (3min each) and mounted with aquamount. Sections were imaged on the Olympus FSX100 microscope 4.2x magnification and images were analysed using Adobe Photoshop CC.

#### 2.2.3 Macrophage abundance

Thawed sections were fixed with paraformaldehyde (4%, 20min), washed in PBS (2 × 5min), incubated in pre-chilled 3% H2O2 in methanol (20min) and then washed in PBS (2 × 5min). Each section was blocked with normal goat serum (NGS, 10%, 30min), incubated with AVIDIN blocking solution (15min), rinsed in PBS and then incubated with rat anti-mouse CD68 primary antibody (1:200, 5% NGS, 4 °C) overnight. The slides were then washed in PBS (2 × 5min) before being incubated with the secondary antibody (1:100, 5% NGS, 30min). Next, the sections were washed in PBS (2 × 5min), incubated with ABC avidin/biotin complex (30min) and DAB solution. Staining reaction was terminated with distilled water. The sections were counterstained with Mayer Haematoxylin for 15sec and rinsed in tap water before blueing in scotts tap water and washing in tap water. Finally, slides were dehydrated in ethanol (95% 3min, 100% 3 × 3min), cleared in xylene (2 × 5min) and mounted with depex. Sections were imaged on the Olympus FSX100 microscope 4.2x magnification and images were analysed using Adobe Photoshop CC.

#### 2.2.4 Collagen

Sections were thawed and fixed in pre-chilled acetone (15min), washed in PBS (2 × 5min), stained in 0.1% Sirius red F3BA (1hr) and then washed in 0.01M HCl (2min). Subsequently, the slides were then dehydrated in alcohol (95%, 5mins; 100%, 2 × 5min), cleared in xylene (2 × 5min) and mounted with depex. Sections were imaged on Olympus BX61 microscope under brightfield and polarised light 4.2x magnification and images were analysed using Adobe Photoshop CC.

### 2.3 *Ex vivo* lipogenesis assay

Primary hepatocytes were isolated by collagenase perfusion [30]. Lipogenesis was determined by measuring the incorporation of [^3^H]acetate or [^14^C]acetate as described [31]. Briefly, cells were cultured for 2 h in serum-free William's E medium before pre-treatment for 1 h with 10 μM metformin or 10 μM O-304, followed by incubation with [^3^H]acetate (5 μCi/mL) and 0.5 mM sodium acetate for 4 h in the continued presence of metformin or O-304. Medium was then removed, and cells washed with PBS before lipid extraction for determination of incorporation into lipid fractions. For cholesterol measurements, the lipid fraction was extracted by homogenizing the hepatocytes in chloroform: methanol (2:1). Lipids were saponified and separated by thin-layer chromatography (TLC) (hexane: diethyl ether: glacial acetic acid = 80:20:1). The tracer incorporated into the cholesterol fraction was measured by liquid scintillation counting and synthesis rates calculated as nmol of ^3^H-labeled acetate incorporated into cholesterol per milligram of protein per hour as described [32].

### 2.4 Metabolic and serum lipid profile measurements

Weekly body weight was determined from 8 weeks of age onward unless otherwise stated. Fed and fasted blood glucose levels were measured using an Accu-Chek Go glucometer. Glucose and insulin tolerance tests were performed on 6-hour or 4-hour fasted mice that were administered intraperitoneally with glucose (1 mg/g body weight) or insulin (0.5 mU/g body weight), respectively. Blood glucose levels were assessed at 0, 15, 30, 60, and 90 min after glucose administration using an Accu-Chek Go glucometer (Roche) as described [33]. Mice were sacrificed by cervical dislocation, and tissues, including liver and white adipose tissue, were weighed and stored for subsequent assays. Trunk blood was collected and centrifuged, and serum was obtained for further analysis. Hepatic and serum cholesterol (total, free, and cholesteryl ester) (Abcam), serum very low-density lipoprotein/low-density lipoprotein (VLDL/LDL), high-density lipoprotein (HDL) cholesterol (Abcam), serum cytokines (TNF, IL1, MCP1, Invitrogen) were measured with commercially available colorimetric kits in accordance with the manufacturers’ specifications.

### 2.5 Lipidomic analysis

#### 2.5.1 Lipid extraction of mouse plasma

Mouse plasma (10 μl) was extracted using a single-phase butanol methanol extraction as previously described [34]. In brief 10 μl of plasma was mixed with 100 μl of a 1-butanol and methanol (1:1 v/v) solution containing 5 mM ammonium formate and a series of internal standard and sonicated for 60 min at 25 °C in a sonic water bath. Immediately after sonication, the mix was centrifuged (16,000×*g*, 10 min, 20 °C). The lipid extracts were then split (40 μl for lipidomic analysis of free cholesterol and cholesteryl esters, 40 μl for oxysterol measurement)

#### 2.5.2 Lipidomic analysis of free cholesterol and cholesteryl esters

Lipidomics was performed as described previously [35]. Analysis of lipid extracts was performed on an Agilent 6490 QQQ mass spectrometer with an Agilent 1290 series HPLC system and a single ZORBAX eclipse plus C18 column (2.1 × 100mm 1.8 mm, Agilent) with the thermostat set at 60 °C. Mass spectrometry analysis was performed in positive ion mode with dynamic scheduled multiple reaction monitoring (MRM) with transitions available at [35].

The running solvent consisted of solvent A: 50% H2O/30% acetonitrile/20% isopropanol (v/v/v) containing 10 mM ammonium formate and solvent B: 1% H2O/9% acetonitrile/90% isopropanol (v/v/v) containing 10 mM ammonium formate. The following mass spectrometer conditions were used: gas temperature, 150 °C, gas flow rate 17L/min, nebulizer 20psi, Sheath gas temperature 200 °C, capillary voltage 3500V and sheath gas flow 10L/min. Isolation widths for Q1 and Q3 were set to “unit” resolution (0.7 amu). Free cholesterol was measured as their in-source fragment (Q1 – 369.3 *m/z*, Q3 – 161.1 *m/z*), while cholesteryl esters were measured with a common product ion of 369.3 *m/z*.

#### 2.5.3 Measurement of oxysterols

To enhance the sensitivity of the low abundant oxysterols, a derivatization was conducted on an aliquot of the lipid extracts. An aliquot of the lipid extract (40 μl) was dried down using a SpeedVac (ThermoFisher) and was subjected to derivatization for oxysterol analysis. To each sample, 200ul of PBS with 96unit/mL of cholesterol oxidase was added and incubated at 37c with shaking for 1 h and 30 min. Subsequently, 500 μl of methanol containing 10 mM of Girard's reagent P (TCI America) and 20 μl of acetic acid. This was then incubated in the dark at room temperature on a shaker. Reaction was quenched with 500 μl of acetone, with vortexing over 15 min. The entire mixture was then dried down again using the SpeedVac, reconstituted with 100 μl of butanol and 100 μl of methanol with sonication in a sonicator bath over 10 min. Samples were centrifuged at 13,000×*g* for 10 min and supernatant transferred into glass vials with inserts for mass spectrometry analysis. Derivative oxysterols were monitored as a neutral loss of 79.3 specific for the derivative group.

### 2.6 Competitive BM transplantation study

WT CD45.1 mice were irradiated (2 × 550rads, 4 h apart) and received BM transplantations the next day with equal portions of WT CD45.1 and WT CD45.2 BM or *Hmgcr KI* CD45.2 BM. Mice were reconstituted for 20 weeks before sacrifice.

### 2.7 Blood leukocytes counts and analysis

Neutrophils, monocytes and monocyte subsets were identified using flow cytometry as previously described [36]. Blood was collected via tail bleeding and collected into EDTA tubes, which were immediately incubated on ice. All subsequent steps were performed on ice. Red blood cells were lysed (BD pharm Lyse; BD Biosciences), and WBCs were centrifuged, washed, and resuspended in HBSS (0.1% BSA w/v, 5 mM EDTA). Cells were stained with a cocktail of antibodies against CD45-PB, Ly6-C/G-PerCP-Cy5.5 (BD Biosciences) and CD115-APC (eBioscience). Monocytes were identified as CD45^hi^CD115^hi^ and further subdivided into Ly6-C^hi^ and Ly6-C^lo^; neutrophils were identified as CD45^hi^CD115^lo^Ly6-C/G^hi^ (Gr-1). Samples were run on the Canto II or LSR Fortessa, and analysed using FlowJo.cBMT study: Blood leukocytes were identified as stated above, with the addition of CD45.1 PECy7 (eBioscience) and CD45.2 APCCy7 (eBioscience) antibodies.

### 2.8 Stem and progenitor cells isolation and analysis

#### 2.8.1 Blood stem and progenitor cells

Blood was harvested and WBCs isolated as described above. Cells were then stained as previously described [36]. Briefly, a cocktail of antibodies to lineage committed cells (CD45R, CD19, CD11b, CD3e, TER-119, CD2, CD8, CD4, and Ly-6G; all FITC; eBioscience) and stem cell markers Sca1-Pacific Blue and ckit-APC-Cy7. HSPCs were identified as lin^–^Sca1^+^ckit^+^. Where further identification of hematopoietic progenitor cells was required, antibodies to CD16/CD32 (FcγRII/III) were used to separate CMPs (lin^–^Sca1^–^ckit^+^FcγRII/III^int^), GMPs (lin^–^Sca1^–^ckit^+^FcγRII/III^hi^) and MEPs (lin^–^Sca1^–^ckit^+^FcγRII/III^lo^). Samples were run on the Canto II or LSR Fortessa, and analysed using FlowJo.

#### 2.8.2 BM stem and progenitor cells

BM was harvested from the femurs and tibias. BM was lysed with RBC lysis buffer (BD pharm Lyse; BD Biosciences), and remaining cells were centrifuged, washed, and resuspended in HBSS (0.1% BSA w/v, 5 mM EDTA) buffer. Stem cell proliferation (cell cycle) was measured using DAPI as per the instruction booklet (Sigma). BM cells were then stained, identified and analysed as stated above. In addition, common beta subunit (CBS; CD131)-PE was also added into the BM stem cell markers antibody cocktail.

cBMT study: BM HSPCs were identified as stated above, with the addition of CD45.1 PECy7 (eBioscience) and CD45.2 AF700 (eBioscience) antibodies.

### 2.9 Splenic leukocytes, HSPCs and progenitor cells analysis

Spleens were dissected from mice and immediately placed on ice. Spleens were flushed through a 40 μm cell strainer with PBS to obtain a single cell suspension. RBCs were lysed, and cells resuspended in HBSS buffer as previously described [36]. Leukocytes, stem cells and progenitor cells were identified and analysed as previous described.

### 2.10 Total and membrane cholesterol content (BODIPY-cholesterol and CTxB) analysis

BODIPY-cholesterol was quantified by flow cytometry. Blood and BM cells were isolated and RBCs lysed as previously described [9]. BM and blood cells were incubated with BODIPY-cholesterol (0.03 mM) for 30min (37 °C) or 1hr (4 °C), respectively. Cells were then washed, and stained with a cocktail of antibodies to identify stem cells or leukocytes, and analysed on FlowJo software as previously described.

Cholera toxin subunit B staining: After single cell suspension, BM and splenic cells were incubated with antibody cocktails to stem cells for 30min. Cells were then washed and stained with Cholera toxin subunit B (CT-B) AF647 at 1:2000 for 30min. Cells were then washed and anti-Cholera toxin subunit B antibody was used to crosslink CT-B-labelled lipid rafts at 1:400 for another 30min. Finally, cells were washed and fixed with 4% PFA for 15min and measured using flow cytometry. FlowJo software was used for analysis.

### 2.11 Statistical analyses

All data are expressed as mean ± SEM. A 2-tailed Student *t* test was used to test differences between 2 groups of mice. Differences among groups of mice were assessed by two-way analysis of variance (ANOVA) or repeated-measures ANOVA. Bonferroni post hoc was performed to identify differences among means. Statistical analyses were assessed using Prism software (GraphPad Software, Inc., LaJolla, CA, USA). Differences were regarded as statistically significant if ∗*P* < 0.05, ∗∗*P* < 0.01, ∗∗∗*P* < 0.001.

## 3 Results

### 3.1 Inhibition of AMPK-HMGCR signalling increases atherogenesis

**Figure 1:**
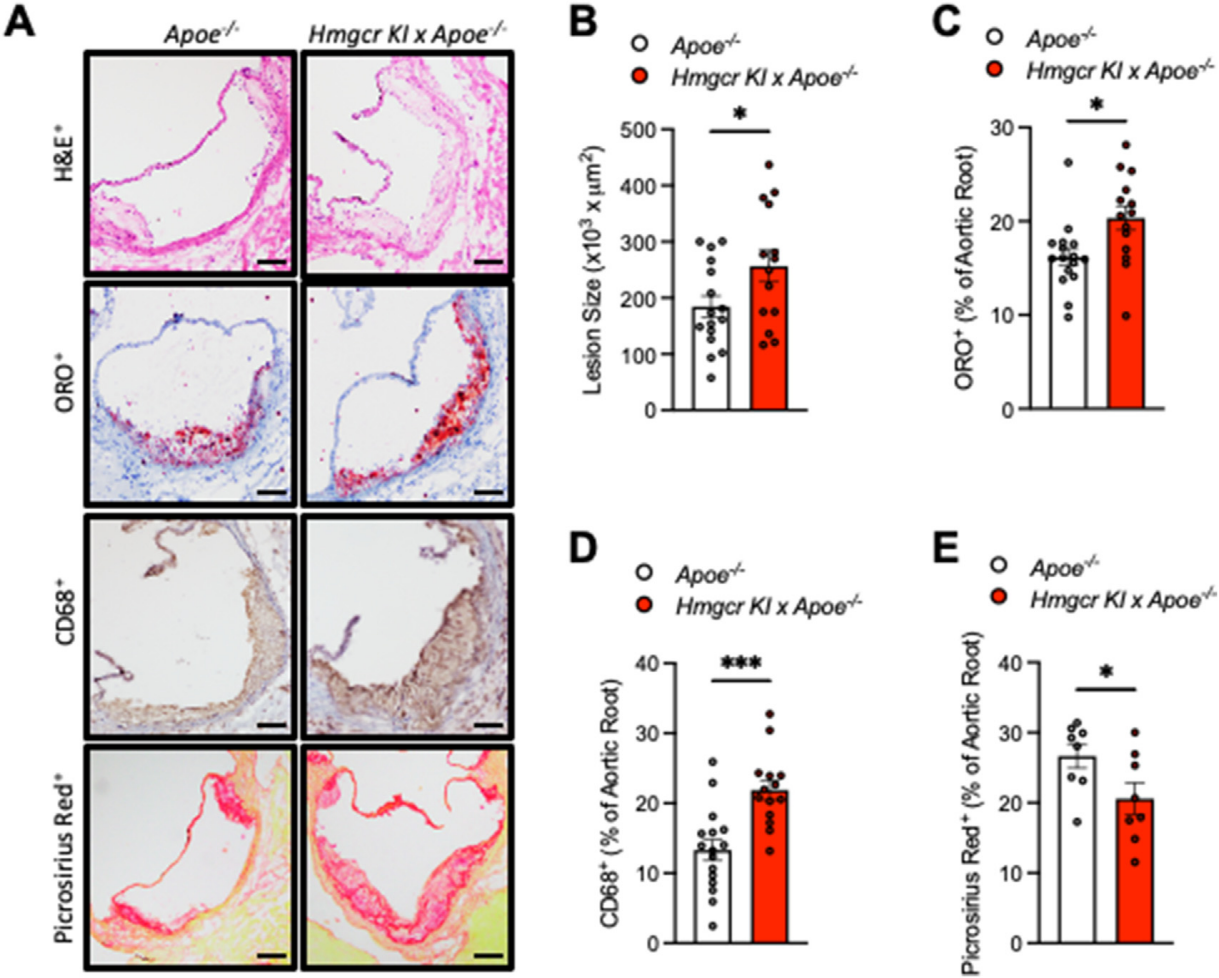
**Inhibition of AMPK-HMGCR signalling increases atherogenesis**. (**A-E**) *Apoe*^*−/−*^ and *Apoe*^*−/−*^*/Hmgcr KI* mice were aged to 20 weeks old on a standard chow diet and aortas were collected to assess for plaque size and composition. In the aortic sinus, (**A**, **B**) lesion size were assessed by hematoxylin and eosin (H&E), (**A**, **C**) lipid content was assessed by Oil Red O (ORO) staining, (**A**, **D**) macrophages were assessed by staining for CD68, (**A**, **E**) collagen content was assessed by staining for picrosirius red. Results shown are a representative image. Scale bars = 50 μM. Data are means ± SEM and analysed using a Student unpaired *t* test, n = 8–16 per group (∗*P* < 0.05, ∗∗∗*P* < 0.001, when comparing *Apoe*^*−/−*^ versus *Apoe*^*−/−*^*/Hmgcr KI*).

To determine the effect of blocking AMPK-HMGCR signalling on atherosclerosis progression, we crossed *Hmgcr KI* mice with atherosclerotic-prone *Apoe* deficient mice to generate *Apoe*^*−/−*^*/Hmgcr KI* double mutant mice. *Apoe*^*−/−*^ mice were used as a control. Mice were aged to 20 weeks old on a standard chow diet to develop atherosclerosis, before the aortic sinus was collected to evaluate atherosclerotic lesion size and plaque composition in male (Figure 1A–E) and female (Figs. S1A–E) mice. While the atherosclerotic lesion size in the control mice corresponded to those expected of *Apoe*^*−/−*^ mice at this age and diet, the cross-sectional lesion areas of the aortic sinuses from *Apoe*^*−/−*^*/Hmgcr KI* were increased by approximately 40% compared to *Apoe*^*−/−*^ control mice (Figure 1A,B). We next characterised the atherosclerotic lesion composition and plaque remodelling by determining lipid, macrophage and collagen content. Interestingly, not only were the lesions found to be larger, but we also observed a significant increase in lipid content in *Apoe*^*−/−*^*/Hmgcr KI* mice as determined by Oil-Red-O staining (Figure 1A,C). Furthermore, in lesions from the aortic sinuses, the areas that positively stained for CD68, a macrophage marker, were also profoundly increased in *Apoe*^*−/−*^*/Hmgcr KI* mice (Figure 1A,D). Moreover, we found that *Apoe*^*−/−*^*/Hmgcr KI* mice exhibited a significant reduction in collagen deposition across their aortic sinus (Figure 1A,E). Importantly, these atherosclerotic plaque phenotypes were also observed in female *Apoe*^*−/−*^*/Hmgcr KI* mice (Figs. S1A–E). Collectively, these data indicate that blocking AMPK signalling to HMGCR not only accelerates the development of atherosclerosis but also renders the atherosclerotic plaque less stable and more susceptible to rupture.

### 3.2 Blocking AMPK signaling to HMGCR leads to increased circulating cholesterol and its metabolites 27-hydroxoysterol levels

**Figure 2:**
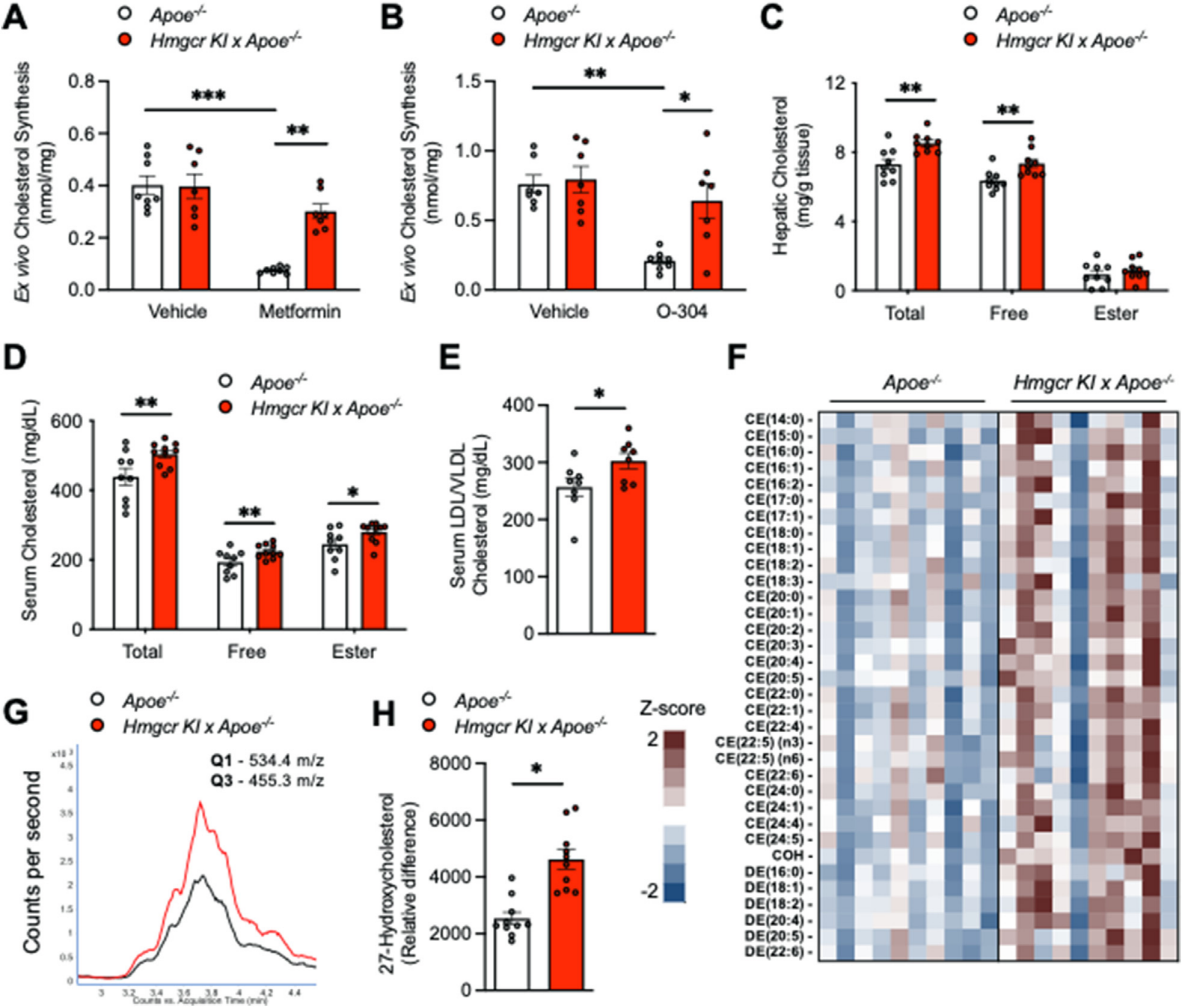
**Elevated circulating cholesterol and its metabolite 27-hydroxoysterol levels in *Apoe*^*−/−*^*/Hmgcr KI* mice**. (**A-B**) Cholesterol synthesis in *Apoe*^*−/−*^ and *Apoe*^*−/−*^*/Hmgcr KI* hepatocytes in response to AMPK activators metformin and O–3O4. n = 3 independent experiments; each experiment contains at least three replicates. (**C-D**) Liver and serum samples were collected from *Apoe*^*−/−*^ and *Apoe*^*−/−*^*/Hmgcr KI* mice. Cholesterol levels, including total cholesterol, free cholesterol, and cholesteryl ester, were measured. (**E**) Serum LDL/VLDL cholesterol from *Apoe*^*−/−*^ and *Apoe*^*−/−*^*/Hmgcr KI* mice. (**F**) Heat map of Z-scored lipid concentrations of species from the cholesteryl ester (CE), free cholesterol (COH) and dehydrocholesteryl ester (DE) classes from *Apoe*^*−/−*^ and *Apoe*^*−/−*^*/Hmgcr KI* mice. (**G**) Overlayed extraction ion chromatogram of the 27-hydroxylated cholesterol measurement for two representative samples. Black trace, *Apoe*^*−/−*^, Red trace, *Apoe*^*−/−*^*/Hmgcr KI.* (**H**) Serum levels of the cholesterol metabolite 27-hydroxycholesterol from *Apoe*^*−/−*^ and *Apoe*^*−/−*^*/Hmgcr KI* mice. Results are means ± SEM and analysed using a two-way ANOVA (in A-D) or a Student *t* test (in E-H), n = 7–10 per group (∗*P* < 0.05, ∗∗*P* < 0.01, ∗∗∗*P* < 0.001, when comparing *Apoe*^*−/−*^ versus *Apoe*^*−/−*^*/Hmgcr KI*).

Our previous studies have shown that the inhibition of AMPK regulation of HMGCR leads to increased hepatic cholesterol synthesis [29]. We next asked if increased hepatic cholesterol synthesis and thus circulating cholesterol levels could also contribute to enhanced atherogenesis in *Apoe*^*−/−*^*/Hmgcr KI* mice. We assessed hepatic cholesterol synthesis *ex vivo* using hepatocytes isolated from control and *Apoe*^*−/−*^*/Hmgcr KI* mice. While the basal rates of synthesis were not different between the genotypes, *Apoe*^*−/−*^*/Hmgcr KI* hepatocytes showed reduced sensitivity to the cholesterol-lowering effects of the AMPK activators metformin and O-304 when compared to vehicle treated hepatocytes (Figure 2A,B). Consistent with this, we found that *Apoe*^*−/−*^*/Hmgcr KI* mice exhibited significant increases in hepatic and serum total and free cholesterol as well as cholesterol esters, the main cholesterol form stored within the cell or exported into the blood stream (Figure 2C,D). Serum concentrations of LDL/VLDL cholesterol were increased in *Apoe*^*−/−*^*/Hmgcr KI* mice (Figure 2E), whereas serum HDL cholesterol levels were comparable between genotypes (Fig. S2A). Similarly, mass spectrometry-based lipidomic analysis revealed a significant increase in serum free cholesterol as well as cholesteryl ester in *Apoe*^*−/−*^*/Hmgcr KI* mice, with comparable triglyceride and diglyceride levels between genotypes (Figure 2F and Figs. S2B–E). More importantly, we identified that the serum levels of the cholesterol metabolite 27-hydroxycholesterol, which is the most abundant oxysterol in circulation and previously found to positively correlate with the development and progression of atherosclerosis both in humans and in rodents [37,38], was also upregulated in *Apoe*^*−/−*^*/Hmgcr KI* mice (Figure 2G–H and Fig. S2F), while serum bile acids remain comparable (Fig. S2G). Consistent with increased atherogenesis, we found that hepatic *Nr1h3* expression and its downstream target genes such as *Srebf1* and *Abca1* mRNA levels were significantly reduced *Apoe*^*−/−*^*/Hmgcr KI* mice (Fig. S2H-M). Given that hyperglycaemia and inflammation are also known to be involved in atherogenesis [39,40], we determined if glucose homeostasis and circulating inflammatory cytokines were altered in *Apoe*^*−/−*^*/Hmgcr KI* mice. We observed no differences in fed and fasted blood glucose levels, whole body glucose tolerance or insulin sensitivity between control and *Apoe*^*−/−*^*/Hmgcr KI* mice (Figs. S3A–C). Similarly, circulating inflammatory cytokines IL-1β, TNF and MCP1 levels were comparable between control and *Apoe*^*−/−*^*/Hmgcr KI* mice (Figs. S3D–F). These data suggest that elevated circulating cholesterol levels and its metabolite, 27-hydroxoysterol, due to loss of HMGCR regulation by AMPK, are associated with enhanced atherogenesis in *Apoe*^*−/−*^*/Hmgcr KI* mice.

### 3.3 Ly6-c^hi^ monocytosis is observed in *Apoe*^*−/−*^*/HMGCR KI* mice

**Figure 3:**
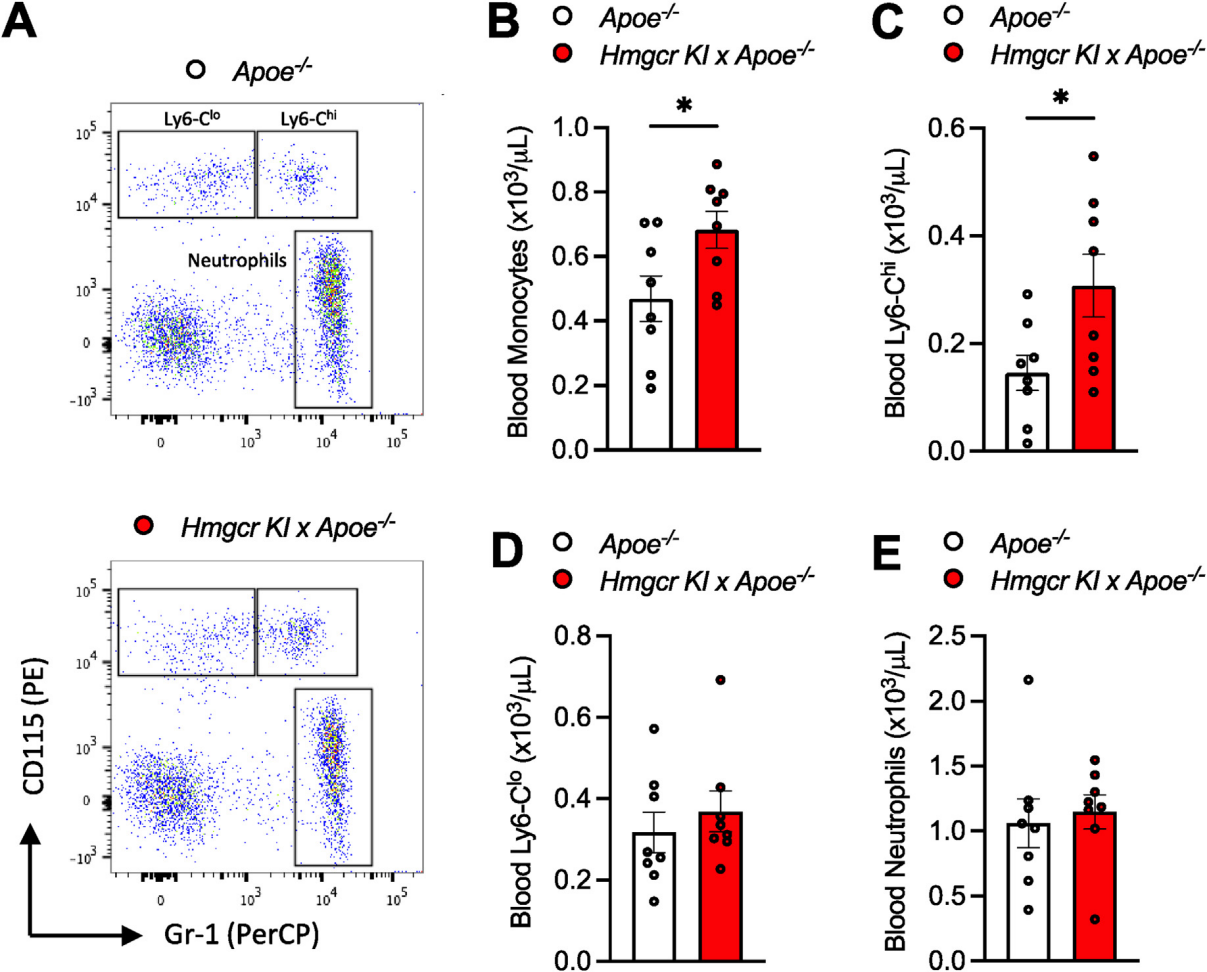
**Increased atherosclerosis in *Apoe*^*−/−*^*/Hmgcr KI* is associated with increased Ly6-C^hi^ monocytosis**. (**A-E**) *Apoe*^*−/−*^ and *Apoe*^*−/−*^*/Hmgcr KI* mice were aged to 20 weeks old on a standard chow diet and blood were collected at the end of the study. Circulating leukocytes were quantified by flow cytometry. (**C**) Ly6-C^hi^ monocytes, (**D**) Ly6-C^lo^ monocytes and (**E**) neutrophil populations were identified and overlayed on the same FACS plot for visualization purpose. Results are means ± SEM and analysed using a Student unpaired *t* test, n = 8 per group (∗*P* < 0.05 when comparing *Apoe*^*−/−*^ versus *Apoe*^*−/−*^*/Hmgcr KI*).

Besides elevated circulating cholesterol levels, circulating monocytes also play a causal role during the development of atherosclerosis. More importantly, defective cholesterol regulation in HSPCs, which are indispensable for the generation of all circulating immune cells, has been shown to contribute to enhanced atherogenesis [10,12,41]. Therefore, we sought to understand whether cholesterol dysregulation via AMPK-HMGCR inactivation could influence myelopoiesis. By assessing circulating leukocytes, we found that *Apoe*^*−/−*^*/Hmgcr KI* mice exhibited increased circulating monocytes, driven by the atherogenic Ly6-C^hi^ subset of monocytes, while neutrophil levels remained comparable between control and *Apoe*^*−/−*^*/Hmgcr KI* mice (Figure 3A–E).

### 3.4 HSPC mobilisation and extramedullary myelopoiesis contribute to monocytosis in the *Apoe*^*−/−*^*/HMGCR KI* mice

**Figure 4:**
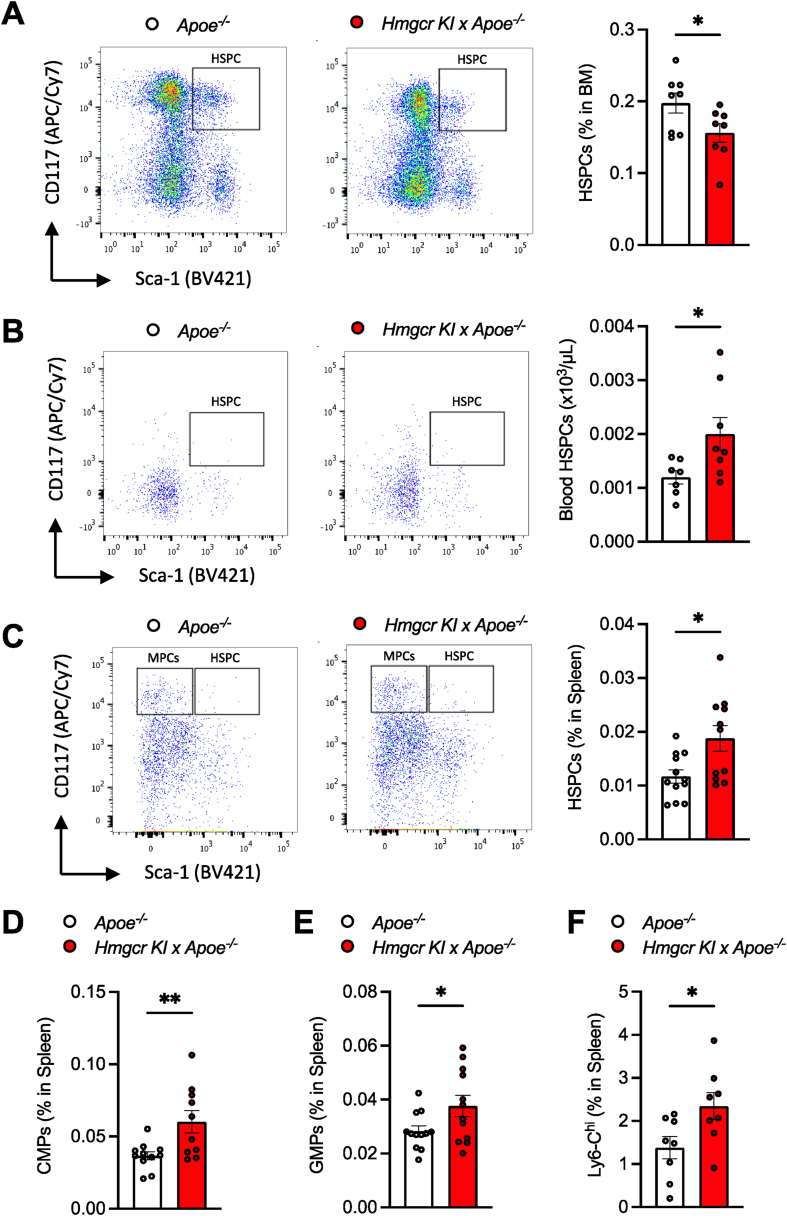
**Inhibition of AMPK-HMGCR signalling promotes HSPC mobilization and extramedullary myelopoiesis**. (**A-F**) *Apoe*^*−/−*^ and *Apoe*^*−/−*^*/Hmgcr KI* mice were aged to 20 weeks old on a standard chow diet. HSPCs in the (**A**) BM, (**B**) blood and (**C**) spleen were measured using flow cytometry. In the spleen, (**D**) CMPs, (**E**) GMPs and (**F**) Ly6-C^hi^ monocytes were measured via flow cytometry. Results are means ± SEM and analysed using a Student unpaired *t* test, n = 7–11 per group (∗*P* < 0.05, ∗∗*P* < 0.01 when comparing *Apoe*^*−/−*^ versus *Apoe*^*−/−*^*/Hmgcr KI*).

To investigate the mechanisms contributing to the increased abundance of circulating monocytes, we explored whether the impaired AMPK-HMGCR pathway may influence myelopoiesis. This was achieved by assessing the number of BM-derived cells from control and *Apoe*^*−/−*^*/Hmgcr KI* mice. Somewhat unexpectedly, we observed a reduction in BM HSPCs in *Apoe*^*−/−*^*/Hmgcr KI* mice (Figure 4A) while BM monocyte and neutrophil counts remained comparable in both genotypes (Figs. S4A–B), suggesting that myelopoiesis did not originate from the BM in *Apoe*^*−/−*^*/Hmgcr KI* mice. Consistent with the reduction in BM HSPCs, flow cytometry analysis revealed a significant increase in the abundance of circulating HSPCs (Figure 4B) and an increase in granulocyte-macrophage (GM)-colony-forming unit (CFU)s (Fig. S4C) in *Apoe*^*−/−*^*/Hmgcr KI* mice, suggesting that HSPCs from the BM had exited the BM and moved into the blood. During atherogenesis, HSPCs mobilise to extramedullary sites such as the spleen, which provides a more permissive microenvironment for proliferation and myeloid differentiation in a process of extramedullary myelopoiesis [12]. In line with this, *Apoe*^*−/−*^*/Hmgcr KI* mice exhibited increased splenic HSPCs (Figure 4C), common myeloid progenitors (CMPs) (Figure 4D) and granulocyte macrophage progenitors (GMPs) (Figure 4E), concomitant with a trend (p = 0.07) in increased spleen weight (Fig. S4D). More importantly, we also found increased splenic Ly6-C^hi^ monocytes by approximately 50% in *Apoe*^*−/−*^*/Hmgcr KI* mice, which have been shown previously to track into atherosclerotic lesions [12] (Figure 4F). Consistent with a selective increase in the Ly6-C^hi^ monocytes, splenic levels of Ly6-C^lo^ monocytes and neutrophils remained comparable (Figs. S4E–F). Similarly, these differences in BM HSPCs, and circulating Ly6-C^hi^ monocytes and HSPCs were also evidenced in female *Apoe*^*−/−*^*/Hmgcr KI* mice (Figs. S5A–D). Collectively, these results suggest that inhibition of AMPK regulation of HMGCR promotes extramedullary myelopoiesis, thereby contributing to the abundance of circulating atherogenic monocytes.

### 3.5 Increased cholesterol accumulation in *Apoe*^*−/−*^*/HMGCR KI* HSPCs contributes to HSPC mobilization and monocytosis

**Figure 5:**
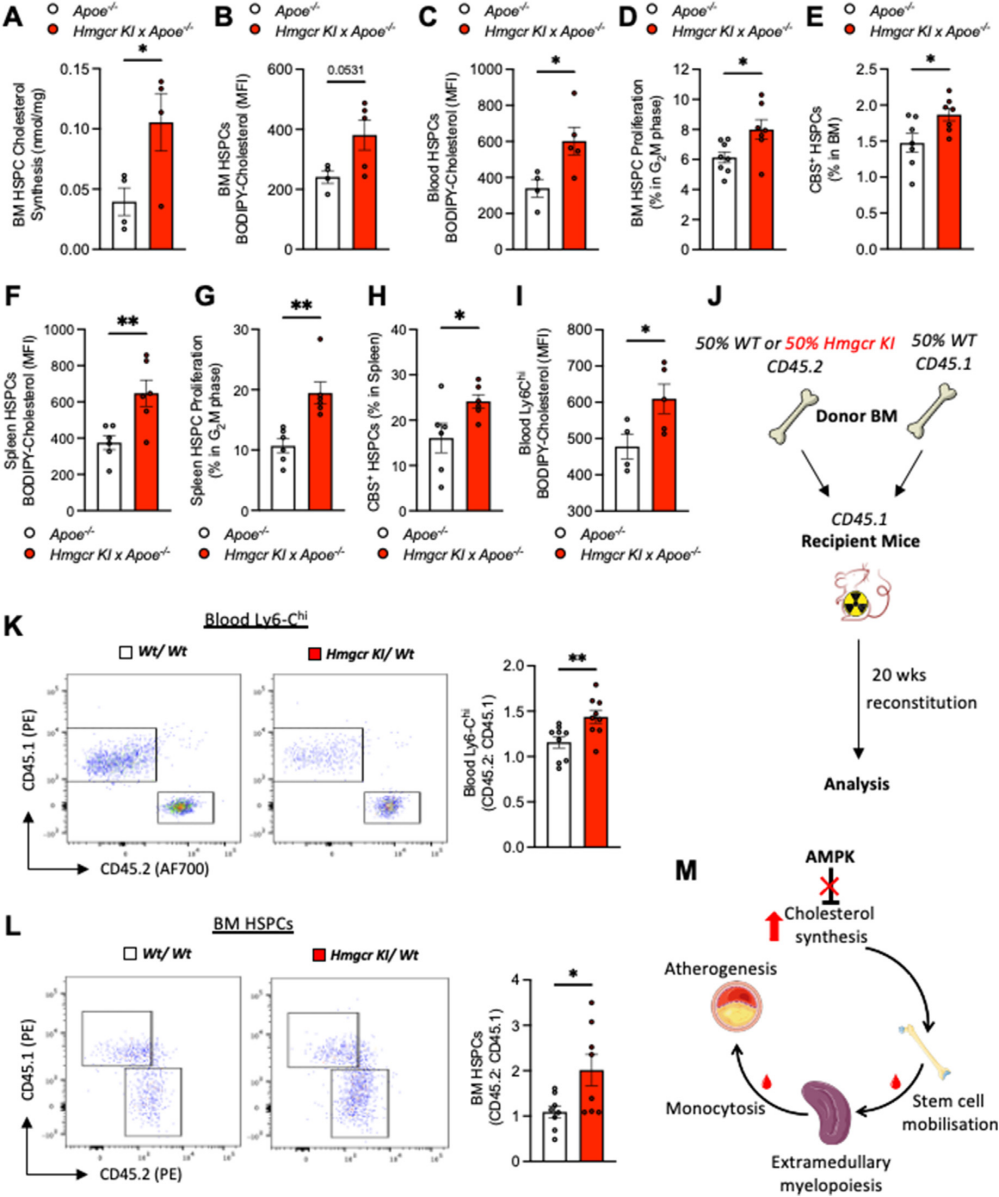
**Increased cholesterol accumulation in *Apoe*^*−/−*^*/HMGCR KI* HSPCs contributes to HSPC mobilization and Ly6-C^hi^ monocytosis**. (**A-I**) *Apoe*^*−/−*^ and *Apoe*^*−/−*^*/Hmgcr KI* mice were aged to 20 weeks old on a standard chow diet. (**A**) BM HSPCs were sorted using FACS before *ex vivo* cholesterol synthesis was measured via ^14^C-acetate labelling and TLC fractionation. n = 4 independent experiments. BODIPY-Cholesterol levels were measured in (**B**) BM HSPCs and (**C**) blood HSPCs. BM HSPC (**D**) proliferation and (**E**) CBS were measured via flow cytometry. BODIPY-Cholesterol levels were measured in (**F**) splenic HSPCs, and (**G**) splenic HSPC proliferation and (**H**) CBS were measured via flow cytometry. BODIPY-Cholesterol levels were measured in (**I**) blood Ly6-C^hi^ monocytes. (**J**) Experimental overview. (**K-L**) Representative flow plots, and ratio of CD45.2 to CD45.1 cells in (**K**) blood Ly6-C^hi^ monocytes and (**L**) BM HSPCs. (**M**) Graphic abstract. Results are means ± SEM and analysed using a Student unpaired *t* test, n = 4–9 per group (∗*P* < 0.05, ∗∗*P* < 0.01 when comparing *Apoe*^*−/−*^ versus *Apoe*^*−/−*^*/Hmgcr KI or Wt/Wt* versus *Hmgcr KI/Wt*).

Previously, we have shown that defective HSPC cholesterol regulation is a key determinant of enhanced myelopoiesis, contributing to increased monocyte production and accelerated atherogenesis [10,12,41]. To understand whether dysregulated HSPC cholesterol synthesis could be contributing to enhanced myelopoiesis in *Apoe*^*−/−*^*/Hmgcr KI* mice, we performed *ex vivo* cholesterol synthesis assay using BM-derived HSPCs isolated from *Apoe*^*−/−*^ and *Apoe*^*−/−*^*/Hmgcr KI* mice. In support of this hypothesis, we found that *Apoe*^*−/−*^*/Hmgcr KI* HSPCs exhibited significant increases in cholesterol synthesis (Figure 5A). More importantly, we also observed an increase in cholesterol content, as shown by the increased staining with BODIPY-cholesterol, in BM- and blood-derived HSPCs of *Apoe*^*−/−*^*/Hmgcr KI* mice (Figure 5B,C). Moreover, membrane cholesterol content as determined by Cholera toxin subunit B (CTxB) staining was also increased in BM-derived HSPCs of *Apoe*^*−/−*^*/Hmgcr KI* mice (Fig. S6A). Interestingly, elevated cellular cholesterol content was associated with increased HSPC proliferation (Figure 5D), concomitant with increased cell surface abundance of the common B subunit (CBS) of the Interleukin 3 (IL-3)/Granulocyte-macrophage colony-stimulating factor (GM-CSF) in the BM-derived HSPCs of *Apoe*^*−/−*^*/Hmgcr KI mice* (Figure 5E)*.* These results suggest that increased cholesterol content in *Apoe*^*−/−*^*/Hmgcr KI* HSPCs may lead to enhanced HSPC expansion and myelopoiesis. Consistently, we observed increased total and membrane cholesterol (Figure 5F and Fig. S6B) and proliferation in splenic HSPCs (Figure 5G–H) and CMPs (Figure 4G–L and Figs. S6C–D) in *Apoe*^*−/−*^*/Hmgcr KI* mice, suggesting that increased monocytosis in *Apoe*^*−/−*^*/Hmgcr KI* mice is driven by extramedullary hematopoiesis in the spleen. Previous studies have shown that increased cholesterol content in mature myeloid cells, including monocytes and macrophages, promotes foam cell formation and contributes to accelerated atherogenesis [42,43]. We hypothesized that this might also be occurring in the mature myeloid cells of *Apoe*^*−/−*^*/Hmgcr KI* mice, which transition to lipid-laden macrophages in the atherosclerotic lesion. Consistent with this, atherogenic monocytes, Ly6-C^hi^ subset from *Apoe*^*−/−*^*/Hmgcr KI* mice exhibited significantly increased cholesterol content (Figure 5I), suggesting that the increased cholesterol content in HSPCs is retained during extramedullary myelopoiesis. To test whether blocking AMPK-HMGCR signalling in HSPCs is responsible for driving extramedullary myelopoiesis independent of circulating cholesterol levels, we performed a competitive BM transplantation (cBMT). This allowed us to assess whether HSPCs with defective AMPK-HMGCR signalling would outcompete wildtype (WT) HSPCs. This was achieved by transplanting equal portions of BM cells from CD45.1 WT mice and either CD45.2 WT or *Hmgcr KI* CD45.2 into lethally irradiated WT recipient mice (Figure 5J). Consistent with our hypothesis, there was significantly greater proportion of circulating *Hmgcr KI* Ly6-C^hi^ monocytes (Figure 5K). Analysis of the BM HSPCs revealed greater proportion of *Hmgcr KI* CD45.2 HSPCs within the BM (Figure 5L), thus suggesting that the *Hmgcr KI* CD45.2 cells have a competitive advantage. Taken together, these results reveal that increased cholesterol accumulation in *Hmgcr KI* HSPCs also intrinsically drives extramedullary myelopoiesis, at least in part advancing atherosclerosis progression.

## 4 Discussion

In the present study, we demonstrated that AMPK regulation of the rate limiting enzyme, HMGCR, in the mevalonate pathway plays a critical role in controlling cholesterol homeostasis in the liver and in HSPCs. Our findings show that blocking AMPK inhibition of HMGCR (*Apoe*^*−/−*^*/Hmgcr KI*) exhibited significantly increased atherosclerotic lesion size combined with elevated lipid and macrophage content. These findings are consistent with previous studies showing that genetic ablation of AMPK α2 subunit increases atherosclerosis [44] and that pharmacological activation of AMPK in atherosclerotic-prone *Apoe*^*−/−*^ mice, with either direct or indirect AMPK activators [[22], [23], [24]], protected against the development and progression of atherosclerosis. More importantly, in line with our previous studies [29], we further showed that in primary hepatocytes isolated from *Apoe*^*−/−*^*/Hmgcr KI* mice, the Ser871Ala mutation rendered HMGCR insensitive to the inhibitory effects of the AMPK activators metformin and O-304 on cholesterol synthesis. It is therefore likely that the increased hepatic cholesterol synthesis which subsequently leads to increased circulating cholesterol and its metabolite 27-hydroxysterol levels was at least in part responsible for the increase in atherosclerotic lesions in *Apoe*^*−/−*^*/Hmgcr KI* mice. However, given that mevalonate pathway products such as geranyl pyrophosphate have been linked to the development of atherosclerosis [45], our study cannot exclude the possibility that other mevalonate pathway products may also contribute to the overall atherogenic phenotypes of *Apoe*^*−/−*^*/Hmgcr KI* mice. Nonetheless, consistent with the single point Ser871Ala mutation, which only alters AMPK signalling to HMGCR rather than affecting AMPK's other functions such as those involved in glucose uptake or fatty acid synthesis, our results show that glucose homeostasis, serum proinflammatory cytokines and triglyceride levels were comparable between control and *Apoe*^*−/−*^*/Hmgcr KI* mice, confirming that increased atherogenesis in *Apoe*^*−/−*^*/Hmgcr KI* mice was unlikely to be due to hyperglycaemia, systemic inflammation or hypertriglyceridemia.

Another possible contributing factor towards enhanced atherogenesis in *Apoe*^*−/−*^*/Hmgcr KI* mice may be a result of extramedullary myelopoiesis. Increased cholesterol accumulation was previously associated with a profound increase in extramedullary myelopoiesis resulting from HSPCs mobilization from the BM, resulting an elevation of circulating atherogenic Ly6-C^hi^ monocyte counts in the blood [[8], [9], [10], [11], [12]]. It has been widely recognised that numbers of circulating Ly6-C^hi^ monocytes that tend to accumulate in the plaques and have the capability to differentiate into pro-inflammatory macrophages, are positively correlated with atherosclerotic progression [[8], [9], [10], [11], [12]]. In agreement with this, *Apoe*^*−/−*^*/Hmgcr KI* mice exhibited significantly increased atherosclerotic plaque macrophage content and circulating Ly6-C^hi^ monocytes concomitant with enhanced blood and splenic HSPCs and myeloid progenitor cells. Our results suggest that increased mobilization of *Hmgcr KI* HSPCs and myelopoiesis were, at least in part, due to increased intracellular HSPC cholesterol content, independent of circulating cholesterol. These findings suggest that, in addition to the liver, the AMPK-HMGCR axis also regulates cholesterol synthesis in HSPCs and that defects in this pathway could be a potential mechanism that is directly responsible for atherogenesis seen in *Apoe*^*−/−*^*/Hmgcr KI* mice. However, given that increased 27-hydroxysterol has been shown to promote myelopoiesis and associated with atherogenesis [37,38,46], it is possible that the increased HSPC mobilization and myelopoiesis in *Apoe*^*−/−*^*/Hmgcr KI* mice could also be attributed to enhanced circulating cholesterol metabolite 27-hydroxysterol. In summary, our findings uncover a fundamental signalling pathway involving the AMPK-HMGCR axis in the regulation of cholesterol homeostasis not only in the liver but also in HSPCs. Inhibition of this regulatory mechanism results in the development and progression of atherosclerosis, suggesting that direct targeting of the AMPK-HMGCR signalling cascade may be an attractive therapeutic option for suppressing the development and progression of atherosclerosis and thus reduce cardiovascular risks. These findings provide a mechanistic basis to support the use of AMPK activators such as salsalate [25], O-304 [47] and PXL 770 [48] for reducing atherosclerotic cardiovascular disease risk.

## Funding

10.13039/501100000925National Health and Medical Research Council of Australia; project grant #1156634 (KL)

10.13039/501100000925National Health and Medical Research Council of Australia; project grant #1085460 (BEK)

10.13039/501100000925National Health and Medical Research Council of Australia; fellowship #1078752 (BEK)

10.13039/501100000925National Health and Medical Research Council of Australia; fellowship #1085752 (AJM)

10.13039/501100000925National Health and Medical Research Council of Australia; project grant #1106154 (AJM)

10.13039/501100000925National Health and Medical Research Council of Australia; project grant #1142938 (AJM)

National Heart Foundation; future leader fellowship #100440 (AJM)

L.E.W Carty Charitable Fund (KL).

CSL Centenary Award (AJM)

Victorian Government's Operational Infrastructure Support Program.

## Author contributions

M.K.S.L designed and performed research experiments, contributed discussion, and edited manuscript. O.D.C, X.L, S.N, D.D, K.H and D.A.O contributed to research experiments and edited manuscript. S.G, P.J.M, T.E, M.D.F and B.E.K contributed discussion and edited manuscript. A.J.M and K.L contributed conceptualization, study design, discussion, wrote and edited manuscript. All authors have read and agreed to the published version of the manuscript.

## Data and materials availability

Further information and requests for reagents may be directed to corresponding authors.

## Materials availability

This study did not generate new unique reagents.

## Data and code availability

This study did not generate any unique datasets or code.
