# Supplementary material for: Defective AMPK regulation of cholesterol metabolism accelerates atherosclerosis by promoting HSPC mobilization and myelopoiesis
Source: Mol Metab. 2022 May 10;61:101514. doi: 10.1016/j.molmet.2022.101514 (PMC9124714; doi:10.1016/j.molmet.2022.101514)
Supplement: Multimedia component 1 [file mmc1.docx]

**Supplementary Materials**

**Supplementary Figures and Figure Legends:**

**Fig. S1. Inhibition of AMPK-HMGCR signalling increases atherogenesis in female mice.**

(**A-E**) Female *Apoe^-/-^* and *Apoe^-/-^/Hmgcr KI* mice were aged to 20 weeks old on a standard chow diet and aortas were collected to assess for plaque size and composition. In the aortic sinus, (**A, B**) lesion size were assessed by hematoxylin and eosin (H&E), (**A, C**) lipid content was assessed by Oil Red O (ORO) staining, (**A, D**) macrophages were assessed by staining for CD68, (**A, E**) collagen content was assessed by staining for picrosirius red. Results shown are a representative image. Scale bars=50 µM. Data are means ± SEM and analysed using a Student unpaired *t* test, n=8-9 per group (**P* < 0.05, ****P* < 0.001, when comparing *Apoe^-/-^* versus *Apoe^-/-^/Hmgcr KI*).

**Fig. S2. Circulating lipid levels in *Apoe^-/-^* and *Apoe^-/-^/Hmgcr KI* mice.** (**A**) Serum HDL cholesterol levels in *Apoe^-/-^* and *Apoe^-/-^/Hmgcr KI* mice. (**B**) Serum free cholesterol, (**C**) cholesteryl ester, (**D**) triacylglycerol, and (**E**) diacylglycerol were measured by mass spectrometry. (**F**) Chromatograms of transition used to measure oxysterols in plasma. Samples were derivatized with Girard's Reagent P prior to analysis. The MRM transition *m/z* 534.4 / 455.3 corresponds to the loss of the derivatized group consistent with a hydroxylated cholesterol. (top graph) chromatogram of derivatized mouse plasma (bottom graph) chromatogram of derivatized 27-hydroxycholesterol standard. (**G**) Serum bile acids were measured using standard commercially available calorimetric kits. (**H-M**) Hepatic *Nr1h3, Srebf1, Abca1, Fasn, Abcg1, Ppara* mRNA levels were examined. Results are means ± SEM and analysed using a Student unpaired *t* test, n = 6-10 per group (**P* < 0.05, when comparing *Apoe^-/-^* versus *Apoe^-/-^/Hmgcr KI*).

**Fig. S3. Glucose homeostasis and circulating inflammatory cytokines in *Apoe^-/-^* and *Apoe^-/-^/Hmgcr KI* mice.** (**A-C**) *Apoe^-/-^* and *Apoe^-/-^/Hmgcr KI* mice were aged to 20 weeks old on a standard chow diet and glucose homeostasis parameters were examined thereafter. (**A**) Fed and overnight fasted blood glucose levels. (**B**) Intraperitoneal glucose tolerance tests (2 g/kg body weight) on 6 h fasted mice treated. Blood glucose levels during glucose tolerance tests were monitored and results are expressed over the time course. (**C**) Mice were fasted 6h and i.p. insulin sensitivity tests (0.75 i.u./kg body weight) were performed, respectively. Blood glucose levels during tolerance tests were monitored and results are expressed over the time course. (**D-F**) Serum MCP-1, IL-1b and TNF-a from *Apoe^-/-^* and *Apoe^-/-^/Hmgcr KI* mice. Data are means ± SEM and analysed using a Student unpaired *t* test, n = 6-11 per group

**Fig. S4. Bone marrow and spleen leukocytes levels in *Apoe^-/-^* and *Apoe^-/-^/Hmgcr KI* mice.** *Apoe^-/-^* and *Apoe^-/-^/Hmgcr KI* mice were aged to 20 weeks old on a standard chow diet and (**A-B**) bone marrow total monocytes and neutrophils were measured and analysed by flow cytometry. (**C**) Granulocyte-macrophage (GM)-colony-forming unit (CFU) assays. (**D**) Spleen weight from *Apoe^-/-^* and *Apoe^-/-^/Hmgcr KI* mice. (**E-F**) Spleen Ly6-C^hi^ monocytes and neutrophils were measured and analysed by flow cytometry. (**G-H**) BODIPY-Cholesterol levels were measured in splenic CMPs and GMPs. (**I-J**) Splenic CMP and GMP proliferation and (**K-L**) CBS were measured via flow cytometry. Results are means ± SEM and analysed using a Student unpaired *t* test, n = 6-8 per group (**P* < 0.05, ***P*<0.01, ****P*<0.001 when comparing *Apoe^-/-^* versus *Apoe^-/-^/Hmgcr KI*).

**Fig. S5. Inhibition of AMPK-HMGCR signalling promotes HSPC mobilization and extramedullary myelopoiesis in female mice.** Female *Apoe^-/-^* and *Apoe^-/-^/Hmgcr KI* mice were aged to 20 weeks old on a standard chow diet and (**A**) blood Ly6-C^hi^ monocytes, (**B**) bone marrow- and (**C**) blood hematopoietic stem and progenitor cells (HSPCs) were measured and analysed by flow cytometry. (**D**) Spleen Ly6-C^hi^ monocytes were measured and analysed by flow cytometry. Results are means ± SEM and analysed using a Student unpaired *t* test, n = 8-9 per group (**P* < 0.05 when comparing *Apoe^-/-^* versus *Apoe^-/-^/Hmgcr KI*).

**Fig. S6. Increased membrane cholesterol content in *Apoe^-/-^/Hmgcr KI* mice.** *Apoe^-/-^* and *Apoe^-/-^/Hmgcr KI* mice were aged to 20 weeks old on a standard chow diet and (**A**) BM and (**B**) splenic HSPCs (**C**) CMPs and (**D**) GMPs were isolated via flow cytometry and stained with Cholera toxin subunit B (CTx-B) for lipid rafts Results are means ± SEM and analysed using a Student unpaired *t* test, n = 5-6 per group (**P* < 0.05, ***P* < 0.01, ****P* < 0.001 when comparing *Apoe^-/-^* versus *Apoe^-/-^/Hmgcr KI*).
